# Saudi Oncology Society clinical management guidelines for testicular germ cell tumors

**DOI:** 10.4103/0974-7796.78551

**Published:** 2011-03

**Authors:** Mohammed Al Otaibi, Mohammed El-naghi, Khaled Balaraj, Shouki Bazarbashi, Dany Rabbah, Khaled Al Othman, Eyad Al Saeed, Abdullah Al Ghamdi, Ali Aljubran, Essam Murshid, Ibraheem Al Oraifi, Hussein Al Kushi

**Affiliations:** Department of Urology, King Faisal Specialist Hospital and Research Center, Riyadh, Saudi Arabia

**Keywords:** Testicular germ cell tumors, Saudi, guidelines, management

## Abstract

In this report, guidelines for the evaluation, medical and surgical management of transitional cell carcinoma of testicular germ cell tumors is presented. It is categorized according to the stage of the disease using the tumor node metastasis staging system, 7^th^ edition. The recommendations are presented with supporting level of evidence.

## INTRODUCTION

Testicular cancer is a rare disease. A total of 38 cases have been diagnosed in 2006, with an age standardized rate (ASR) of 0.5 cases per 100,000 representing 1% of all diagnosed cancer in Saudi Arabia (www.scr.org.sa)

Due to the rarity of the disease and the need for multidisciplinary approach in managing testis cancer the group recommended that ‘All testicular cancer cases should be managed in tertiary care centers.’

A panel of experts in the management of testicular cancer was gathered under the umbrella of the Saudi Oncology Society. It included Urologists, Medical oncologists and Radiation oncologists. A subgroup was formed to work on testes cancer. The subgroup reviewed the literature, current international guidelines in testicular cancer management. The subgroup brought their recommendation to the panel where all references were discussed in several meetings and the guidelines were finalized.

We have used the following evidence level:

- (EL1) High level: well-conducted phase III randomized trials or meta-analysis.
- (EL2) intermediate level: good phase II trials or phase III with limitations.
- (EL3) low level: Observational/retrospective studies or expert opinion.

## 1. STAGING

The American Joint Committee on Cancer (AJCC) TNM staging for testis cancer (7 ^th^edition 2010) was used.

## 2. RISK STRATIFICATION

The international Germ Cell Cancer Collaborative Group Risk Classification[1] should be used:

**Table:** 

2.1.	Good prognosis
2.1.1.	For patients with seminoma:
2.1.1.1.	Any primary site
2.1.1.2.	No non-pulmonary visceral metastasis
2.1.1.3.	Normal serum AFP, any serum beta-hCG or LDH
2.1.2.	For patients with non-seminoma (NSGCT):
2.1.2.1.	Testicular or retroperitoneal primary tumor
2.1.2.2.	No non-pulmonary visceral metastasis
2.1.2.3.	Serum AFP less than 1000 ng/mL, beta-hCG less than 5000 mIU/mL and LDH less than 1.5 times the upper limit of normal
2.2.	Intermediate prognosis:
2.2.1.	For patients with seminoma:
2.2.1.1.	Any primary site
2.2.1.2.	Non-pulmonary visceral metastasis
2.2.1.3.	Normal serum AFP, any beta-hCG or LDH
2.2.2.	For patients with NSGCT:
2.2.2.1.	Testicular or retroperitoneal primary
2.2.2.2.	No non-pulmonary visceral metastasis
2.2.2.3.	Any of the following: serum AFP 1000 to 10,000 ng/mL; beta-hCG 5000 to 50,000 mIU/mL; LDH 1.5 to 10 times upper limit of normal
2.3.	Poor prognosis:
2.3.1.	For NSGCT only, any of the following:
2.3.1.1.	Mediastinal primary site
2.3.1.2.	Non-pulmonary visceral metastasis
2.3.1.3.	Serum AFP >10,000 ng/mL; serum beta-hCG >50,000 mIU/mL; LDH more than 10 times upper limit of normal

## 3. TREATMENT

Will depend on the histological subtype as follows:

**Table:** 

3.1.	Seminoma: All stages should undergo urgent inguinal orchiectomy. Trans-scrotal biopsy or orchiectomy for any intra-testicular lesion is absolutely contra-indicated. Further treatment will depend on the stage:
3.1.1.	Stage I: One of the following adjuvant options:
3.1.1.1.	Chemotherapy: single agent carboplatin: 1–2 doses at AUC 7.[2](EL 1)
3.1.1.2.	Radiotherapy: infradiaphragmatic para-aortic ± ipsilateral iliac nodes.[3,4] (EL1)
3.1.1.3.	Surveillance: this should be done only in compliant patients with primary tumors less than 4 cm and less than pT2.[5] (EL1)
3.1.2.	Stage IIA and IIB:
3.1.2.1.	Radiotherapy to infradiaphragmatic para-aortic and ipsilateral Iliac nodes.[6] (EL2)
3.1.2.2.	For selected stage IIB, chemotherapy with four cycles of EP (Etoposide and cisplatin) or three cycles of BEP (bleomycin, etoposide and cisplatin). (EL2)
3.1.3.	Stage IIC and III: treatment will depend on the risk classification:
3.1.3.1.	Good risk: chemotherapy with four cycles of EP (for patients with compromised lung function), or three cycles of BEP.[7–8] (EL1)
3.1.3.2.	Intermediate risk: chemotherapy with four cycles of BEP.[9] (EL1)
3.1.4.	Management of post-chemotherapy residual nodes/masses seen on CT scan: this depends on the size and the level of tumor marker. (HCG)
3.1.4.1.	If size less than 3 cm and normal markers: surveillance
3.1.4.2.	If more than 3 cm and normal markers: do PET scan:[10]
3.1.4.2.1.	If negative: surveillance. (EL2)
3.1.4.2.2.	If positive consider one of the following options:
3.1.4.2.2.1.	Surgical resection
3.1.4.2.2.2.	Biopsy and second-line chemotherapy if positive for residual disease (See item 3.2.5.3.2)
3.1.4.2.2.3.	Radiotherapy
3.1.4.3.	If the residual mass is enlarging or markers increasing: second-line chemotherapy (EL2) - See item 3.2.5.3.2
3.1.5.	Management of patients failing first line chemotherapy: patients will receive second line chemotherapy; options are:
3.1.5.1.	Four cycles of VeIP regimen (Vinblastine, Ifosfamide and cisplatin).[11] (EL2) or
3.1.5.2.	Four cycles of TIP regimen (paclitaxel, Ifosfamide and cisplatin).[12] (EL 2)
3.1.6.	Management of patients failing second line chemotherapy: patients will be treated with combination paclitaxel and Gemcitabine for those who did not receive paclitaxel before.[13]
3.2.	Non-seminoma: all stages will undergo urgent inguinal orchiectomy. Trans-scrotal biopsy or orchiectomy for any intra-testicular lesion is absolutely contra-indicated. Further treatment will depend on the stage as follows:
3.2.1.	Stage I:
3.2.1.1.	Treatment will depend on the presence of any the following risk factors:[14]
3.2.1.1.1.	Lymphovascular invasion
3.2.1.1.2.	Presence of embryonal histology (50% or more)[15]
3.2.1.1.3.	Absence of yolk sac histology
3.2.1.1.4.	Tumor stage more than T1
3.2.1.2.	Stage I with no risk factors; options are:
3.2.1.2.1.	Surveillance: should be reserved in compliant patients.[16–17] (EL2)
3.2.1.2.2.	Two cycles of adjuvant chemotherapy with BEP regimen.[16–18] (EL1)
3.2.1.2.3.	Open nerve sparing retroperitoneal lymph node dissection: to be done only in high-volume tertiary care centers[18] (EL2): further therapy will depend on the pathological result as follows:
3.2.1.2.3.1.	pN0: surveillance
3.2.1.2.3.2.	pN1: surveillance in compliant patients or two cycles of chemotherapy with BEP in non-compliant patients. (EL3)
3.2.1.2.3.3.	pN2: three cycles of chemotherapy with BEP regimen. (EL3)
3.2.1.2.3.4.	pN3: three cycles of chemotherapy with BEP regimen. (EL3)
3.2.1.3.	Stage I with any risk factor of above; options are:
3.2.1.3.1.	two cycles of adjuvant chemotherapy with BEP regimen[16]
3.2.1.3.2.	Open nerve sparing retroperitoneal lymph node dissection (RPLND): to be done only in high-volume tertiary care centers[19] (EL2): further therapy will depend on the pathological stage as in item 3.2.1.2.3
3.2.1.4.	Stage Is: patient should receive three cycles of systemic chemotherapy with the BEP regimen. (EL3)
3.2.2.	Stage IIA and IIB: options of therapy will depend if markers (AFP and HCG) are normal or elevated:
3.2.2.1.	Normal markers; options are:
3.2.2.1.1.	Primary chemotherapy with three cycles of BEP[8]
3.2.2.1.2.	Open nerve sparing RPLND,[20–21] if the nodal metastasis is in the primary landing zone. Further therapy will depend on the pathological stage as in item 3.2.1.1.3
3.2.2.2.	Elevated markers: systemic chemotherapy depending on the international risk classification group
3.2.2.2.1.	Low risk: three cycles of BEP[7–8]
3.2.2.2.2.	Intermediate and high risk: four cycles of BEP9
3.2.3.	Stage IIC and III: treatment will be with chemotherapy depending on the international risk classification:
3.2.3.1.	Low risk: three cycles of BEP chemotherapy[7–8]
3.2.3.2.	Intermediate and high risk: four cycles of BEP chemotherapy[9]
3.2.4.	Management of post chemotherapy:
3.2.4.1.	No residual disease and normal markers: surveillance[22]
3.2.4.2.	No residual disease and elevated markers (AFP and HCG): Second-line chemotherapy. See item 3.2.4.3.2
3.2.4.3.	Residual disease by CT scan: this depend on the level of serum markers:
3.2.4.3.1.	Normal markers: RPLND and resection of all residual disease if technically feasible:[23–24] further therapy will depend on pathology result:
3.2.4.3.1.1.	Mature teratoma, necrosis or fibrosis: no further therapy
3.2.4.3.1.2.	Residual germ cell tumor: two cycles of chemotherapy[25] with EP, VIP or TIP (see below) (EL2)
3.2.4.3.2.	Elevated markers: second line chemotherapy; options are:
3.2.4.3.2.1.	Four cycles of VeIP regimen[11]
3.2.4.3.2.2.	Four cycles of TIP regimen[12]
3.2.5.	Management of patients failing second line chemotherapy: patients will be treated with paclitaxel and Gemcitabine if they did not receive paclitaxel before[13]
3.2.6.	Management of patients failing all lines of chemotherapy: In the case of markers progression after salvage treatment and exhaustion of all possible chemotherapeutic options, resection of residual tumors (desperation surgery) should be considered if complete resection of all tumors seems technically feasible.[26]
